# The identification of risk factors contributing to accidental opioid poisonings in companion dogs using data from a North American poison control center (2006-2014)

**DOI:** 10.1371/journal.pone.0227701

**Published:** 2020-01-29

**Authors:** Mohammad Howard-Azzeh, David L. Pearl, Terri L. O’Sullivan, Olaf Berke

**Affiliations:** Department of Population Medicine, Ontario Veterinary College, University of Guelph, Guelph, Ontario, Canada; University of Lincoln, UNITED KINGDOM

## Abstract

In the last decade, there has been a marked increase in opioid-related human deaths in the U.S. However, the effects of the growth in opioid use on vulnerable populations, such as pet dogs, are largely unknown. The objective of this study was to investigate potential risk factors at the dog, county, and state-levels that contributed to accidental dog opioid poisonings. Dog demographic information was collected during calls to the Animal Poison Control Center (APCC), operated by the American Society for the Prevention of Cruelty to Animals, about pet dog exposures to poisons from 2006–2014. Data concerning state-level opioid-related human death rates and county-level human opioid prescription rates were collected from databases accessed from the Centers for Disease Control and Prevention. A multilevel logistic regression model with random intercepts for county and state was fitted to explore associations between the odds of a call to the APCC being related to dog opioid poisonings with the following independent variables: sex, weight, age, reproductive status, breed class, year, source of calls, county-level human opioid prescription rate, and state-level opioid human death rate. There was a significant non-linear positive association between accidental opioid dog poisoning calls and county-level human opioid prescription rates. Similarly, the odds of a call being related to an opioid poisoning significantly declined over the study period. Depending on the breed class, the odds of a call being related to an opioid poisoning event were generally lower for older and heavier dogs. The odds of a call being related to an opioid poisoning were significantly higher for intact compared to neutered dogs, and if the call was made by a veterinarian compared to a member of the public. Veterinarians responding to poisonings may benefit from knowledge of trends in the use and abuse of both legal and illegal drugs in human populations.

## Introduction

Every year, more lives are claimed by drug use disorders in the U.S., with opioids contributing to approximately 72% of all drug use related deaths [1]. In 2015, the abuse of both legal and illegal opioids caused roughly 122,000 deaths globally, of which 33,000 deaths occurred in the U.S. alone [1–4]. Opioids are the second most commonly abused illicit drug in the U.S. after cannabis [5].

Recent studies investigated the impact of adult opioid use on accidental opioid exposures in minors and a clear association has been reported between adult opioid use and accidental opioid poisonings in children within the same household [1,6–8]. As a result of increasing adult use of opioids, hospitalizations caused by opioid poisonings have increased almost two-fold in the pediatric population from 1997–2012 [9].

Like young children, dogs are also extremely curious, and thus vulnerable to the effects of human drug use. This is important because opioid overdoses in both dogs and children cause a wide range of negative cardiovascular and neurological health effects, which can be fatal without timely intervention [10]. Yet, little has been published on the impact of human opioid use on dogs and few cases have been reported in the veterinary literature. The likelihood of accidental marijuana poisonings in both children and dogs are associated with adult marijuana use patterns, and adult use of other drugs, may also result in accidental exposure to these populations [11,12]. Furthermore, it has been shown that various dog characteristics, such as sex and breed, may be associated with accidental poisonings from other substances such as insecticides and toxic plants [13]. These results suggest human use patterns and dog-level characteristics may be associated with opioid poisoning in dogs. Consequently, the objectives of this study were to identify the impact of human opioid use patterns and dog-level characteristics on the odds of a call to an animal poison control center being related to an opioid poisoning in the U.S.

## Methods

### Data

The American Society for the Prevention of Cruelty to Animals (ASPCA) operates the Animal Poison Control Center (APCC), an emergency poison control hotline that gives toxicological advice to the public, veterinarians, and other poison control centers that are administering care to a potentially poisoned animal. The APCC collects data from each call regarding the number of animals exposed, toxicant, patient characteristics, clinical effects, outcome, and date/location/time of call. These data are stored in the APCC's AnTox toxicology database. AnTox data collected between 2006–2014 were used in this study. Although the APCC receives calls from Canada, only calls from the U.S. were used in this study. Assistance from the APCC costs 65 USD and the services can be used by veterinarians and members of the public whenever it is required.

Each call to the APCC concerning a dog patient was considered an observation. The data obtained from the AnTox database during the study period included 189,594 unique observations concerning poisonings of individual dogs. The variables used in this study from each observation were the following dog-level characteristics: weight (kg), breed, age (years), reproductive status, sex, toxicant exposure, call source, year, and the latitude/longitude of the call's location. The location data were used to identify the county and state of each call. A case was defined as any call from the AnTox database that involved a dog exposed to an opioid, regardless of route of exposure. These opioids included all prescription and illicit opioids as well as over the counter drugs containing opioids (e.g., loperamide) that can be abused. If a dog was exposed to an opioid and another toxicant at the time of the call, it was also considered a case. A control was a call involving a dog from the AnTox database that was exposed to any non-opioid toxicant. Multiple calls to the APCC concerning the same poisoning event were treated as a single call. The source of call is noted in the AnTox database and includes the following categories: public, veterinarian, not asked, other poison control center, unknown, and Animal Product Safety Service. Only calls from the public and veterinarians were included in this study. In reviewing age and weight variables, observations were treated as missing data if "0" or an unrealistic value was recorded. For instance, weights exceeding 114 kg for giant breed dogs (Mastiffs, Neapolitan Mastiffs, Newfoundlands, Tibetan Mastiffs, Leonbergers, Boerboels, St. Bernards, Great Danes) or exceeding 75 kg for all other breeds were considered implausible. Similarly, data from dogs with their age recorded as greater than 26 years old were not used in this study.

Based on the primary breed assigned to each dog in the APCC database, dogs were assigned to the following American Kennel Club (AKC) breed classes: herding, hound, non-sporting, sporting, terrier, toy, working, Foundation Stock Service (FSS), and other. A small number of dogs (n = 91) whose breeds fell under AKC’s miscellaneous category were re-classified as part of the FSS category. Dog breeds that are not recognized by the AKC were classified into the “other” category. The AnTox database contains data reporting the breed of a dog being mixed, pure, or if the owners were not asked. In 74% of calls, this field was “not asked”, so the main/apparent breed was used to classify the dog to its breed class.

The original AnTox coding of the sex variable was male, female, did not ask, group, and unknown. Only male and female was used in this study. The reproductive status variable was originally coded in the AnTox database as immature, intact, lactating, neutered, pregnant, or unknown. These data points were used to determine if animals were intact or neutered for subsequent analyses.

County-level human opioid prescription rate data were collected from Centers for Disease Control and Prevention’s (CDC) analysis of data from IQVIA Xponent's database from 2006 to 2014 [14]. Prescription rates for each county were reported as prescriptions per 100 people per year. Prescription rate data were unavailable from counties for 7.2% of APCC canine related calls. The data included both initial and refill prescriptions paid for by commercial insurance, Medicaid, Medicare, or cash or its equivalent and dispensed at a retail pharmacy. Prescription rate data are approximated from a sample of 50,000 non-hospital pharmacies that dispense roughly 90% of all initial and refill U.S. retail prescriptions. State-level opioid-related human death rates were collected from CDC’s WONDER Online Database from 2006 to 2014 [15]. Opioid deaths were identified from the WONDER Database using the International Classification of Diseases, 10th Revision (ICD–10) multiple-cause-of-death codes: T40.1-T40.4. Death rates were reported as deaths per 100,000 people per year. To estimate prescription rates, the CDC used annual resident population denominator estimates obtained from the U.S. Census Bureau. Data pertaining to state-level death rates were missing for North Dakota for 2006 and 2012.

### Statistical analysis

Data (n = 189,594) were analyzed using Stata 15 (StataCorp, College Station, TX). Descriptive statistics, including the proportion of opioid-related calls versus all other calls for each year, were performed. To account for clustering throughout the statistical modeling process, hierarchical random intercepts for county and state were added in all univariable and multivariable models. For calls representing a household with several dog poisonings, only one randomly chosen animal was included in the analysis to avoid model convergence problems that occurred from including a random intercept for household.

Univariable mixed logistic regression models were fitted to assess the association between the independent variables and the log odds of a dog poisoning being related to an opioid exposure. A liberal significance level (α = 0.20) was used to identify variables for inclusion in a multivariable model. To avoid issues associated with collinearity, we examined the correlation between independent variables with various correlation coefficients (i.e., Spearman’s rank, Pearson, and Phi coefficient) depending on the form of the independent variables. If the correlation between two variables were greater than |0.75|, only the more biologically plausible variable was considered for inclusion in the multivariable model. For continuous independent variables, linearity between the predictor variable and the log odds of being an opioid-related call was assessed graphically using locally weighted scatterplot smoothing (LOWESS) curves. If the relationship between the predictor variable and the log odds of being an opioid-related call was not linear, then a quadratic term for that predictor variable was generated, and the two terms were fitted together. If the p-value of the quadratic term was less than 0.05, and it was appropriate to model as a quadratic relationship, based on the LOWESS curve, then the quadratic relationship was explored in subsequent multivariable modeling.

**Fig 1:**
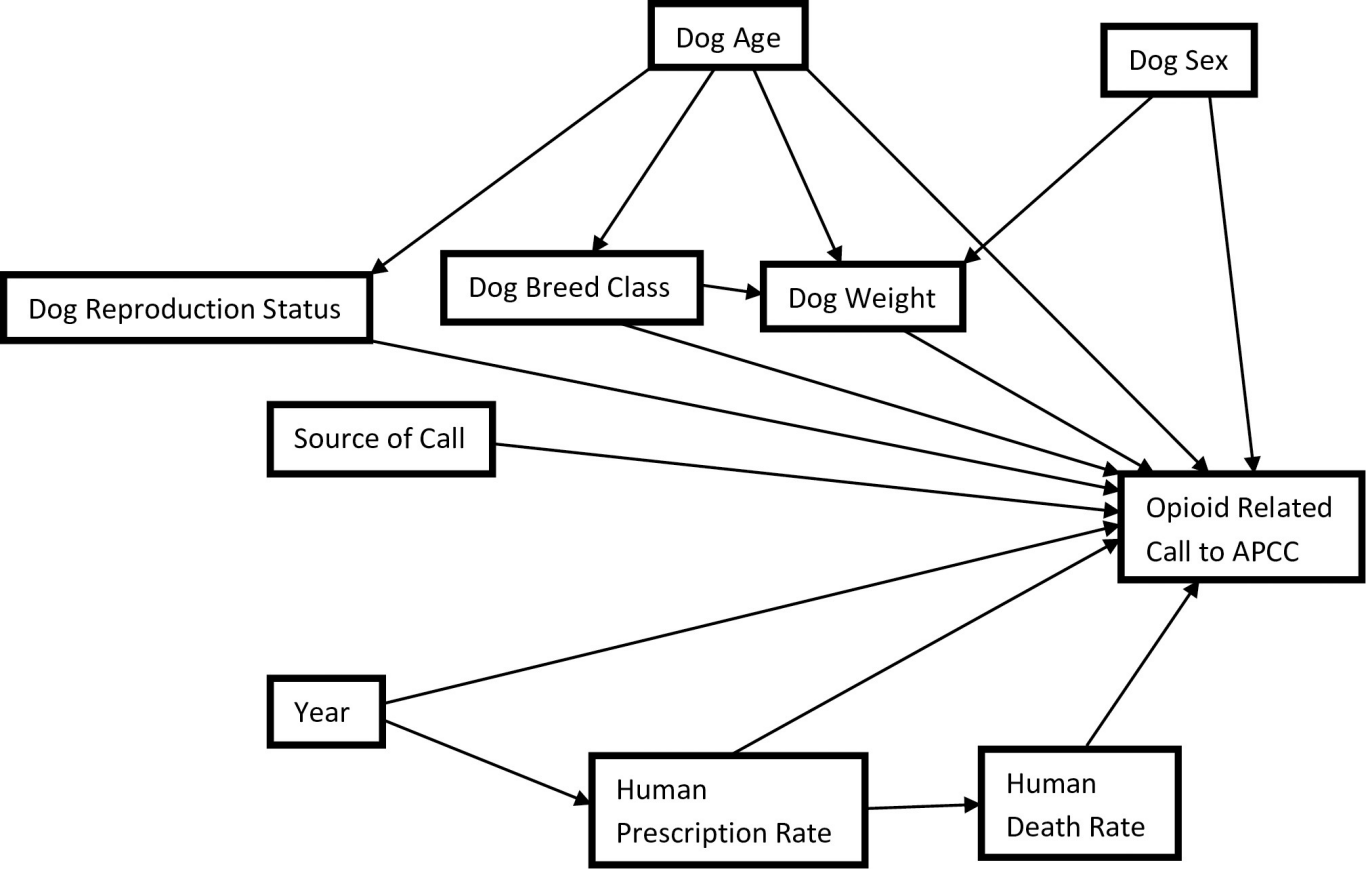
Causal diagram depicting the relationship between dog-level and community-level factors and the odds of a call about an animal being related to intoxication with an opioid.

To fit mixed logistic regression models, a manual forward selection process was applied. Each possible predictor variable was added one at a time from most to least significant based on univariable analyses. Two-way dog-level (age, sex, reproductive status, breed, and weight) and state/county-level (death rate, prescription rate, and year) interactions that were identified a-priori to be biologically plausible were assessed one at a time in a main effects model. Interactions with a p-value of less than 0.05 were kept in the model. A global Wald's χ^2^ test was used to examine the significance of variables with more than two categories. Any variable that caused 30% or greater change in the coefficient of another significant variable on its removal from the model was considered an explanatory antecedent (i.e., confounder) assuming it met the causal criteria (i.e., non-intervening variable) based on our causal diagram (Fig 1). Variables were included in the final model if they had a p-value of less than 0.05, were part of a significant interaction term, or acted as an explanatory antecedent. Pearson and deviance residuals were assessed for outliers at the dog-level, while the normality and homoscedasticity of the best linear unbiased predictors were assessed at the county and state-levels. The variance components from the final model were used to estimate the variance partition coefficients (VPCs) at the dog, county, and state-levels using the latent variable technique [16].

In light of recent concerns over the misuse of the phrase "statistically significant" [17], we have provided the following disclaimer. In this manuscript, the term “statistically significant” is not intended to infer biological or epidemiological importance, or causation. However, it is used to denote when we have sufficient evidence based on our statistical criteria to suggest that the measure of association for a particular variable or contrast is different from the null value [17].

## Results

### Descriptive statistics

The frequencies of observations for categorical variables were relatively evenly distributed across all categories with few exceptions (Table 1). Calls to the APCC were mainly made by the public, while most calls concerned sporting and toy breeds (Table 1). The distribution of male and female dogs was similar, but the majority of dogs were neutered (Table 1). The median age of dogs was 2 years, and the median weight of dogs was 12.1 kg, with interquartile ranges (IQR) of 0.9 to 6 years old and 5.8 to 25.5 kg, respectively (Table 2). The median county prescription rate was 68.5 prescriptions per 100 people per year with an IQR of 50.7 to 83.9 prescriptions per 100 people per year (Table 2). The median opioid-related state death rate was 6.8 deaths per 100,000 people per year with an IQR of 5.1 to 8.8 deaths per 100,000 people per year (Table 2).

**Table 1:** Frequency of U.S. dogs reported to the APCC^ǁ^ (2006–2014).

Parameter	Frequency	Percentage of dataset
**Sex**		
Female	97,834	51.60
Male	91,760	48.40
**Reproductive Status**		
Intact	41,297	21.78
Neutered	142,643	75.24
Unknown	5,654	2.98
**Breed Class**^†^		
Herding	15,969	8.42
Hound	17,051	8.99
Non-Sporting	15,881	8.38
Sporting	44,381	23.41
Terrier	20,558	10.84
Toy	46,572	24.56
Working	16,543	8.73
Foundation Stock Service	528	0.28
Other^‡^	12,111	6.39
**Source of Call**		
Public	133,148	70.46
Veterinarian	56,432	29.76

^ǁ^Animal Poison Control Center

^†^Breed classes as defined by the American Kennel Club

^‡^Breeds in AnTox database that are not delineated into American Kennel Club defined breed classes

**Table 2:** Statistics describing the age and weight of U.S. dogs reported to the APCC^ǁ^, and human opioid death/prescription rates in their states or counties, respectively (2006–2014).

Parameter	Mean	Median	Standard Deviation	Interquartile Range	N^‡^
**Dog Age (years)**	3.7	2.0	3.5	0.9–6.0	189,584
**Dog Weight (Kg)**	16.4	12.1	12.6	5.8–25.5	189,584
**Human Opioid Death Rate**^†^ **(Deaths per 100,000 per year by state)**	7.3	6.8	3.0	5.1–8.8	51
**Human Opioid Prescription Rate**^†^ **(Prescriptions per 100 people per year by county)**	70.2	68.5	26.0	50.7–83.9	2,238

^ǁ^Animal Poison Control Center

^†^Data collected from the Centers for Disease Control and Prevention

^‡^Total number of dogs, states, or counties depending on the variable

From 2006 to 2014, 2.72% (n = 5,162) of calls concerning dogs were related to an opioid exposure (Table 3). Although calls to the APCC consistently increased over the period of the study, with the APCC receiving 16,663 calls in 2006 and 22,833 in 2014, the proportion of opioid poisoning calls by year was variable. From the beginning of the study in 2006, the proportion of opioid poisoning calls increased until it peaked in 2008 at 3.26% of all calls. After 2008, the proportion of opioid-related calls consistently decreased until it reached its lowest level (2.17%) at the end of the study period.

**Table 3:** Statistics describing the frequency of opioid and non-opioid calls on behalf of U.S. dogs to the APCC^ǁ^ from each year of the study (2006–2014).

Call Type	Year									
	2006	2007	2008	2009	2010	2011	2012	2013	2014	Total
**Non-Opioid**	16,663	19,282	20,435	20,277	20,724	20,963	21,129	22,126	22,833	184,432
**Opioid**	498	614	689	657	609	574	512	503	506	5,162
**Percent Opioid calls**	2.90%	3.1%	3.26%	3.08%	2.85%	2.67%	2.37%	2.27%	2.17%	2.72%
**Total**	17,161	19,896	21,124	20,934	21,333	21,537	21,641	22,629	23,339	189,594

^ǁ^Animal Poison Control Center

### Bivariate relations

All independent variables examined were statistically significant (i.e., P≤0.20) based on our univariable mixed logistic regression model (Table 4). However, during forward model building, state-level opioid-related human death rate did not meet the inclusion criteria (i.e., p≤0.05) required for inclusion in the final multivariable mixed logistic regression model.

**Table 4:** Final model^†^ examining the associations between each individual dog-level and community level variable on the odds of a U.S. dog poisoning call to the APCC^ǁ^ being related to an opioid (2006–2014).

Parameter	Coefficient	Coefficient 95% CI	P-Value
**Age**	-0.0909	-0.100; -0.0814	<0.001
**Age**^**2**^	0.0196	0.0177; 0.0215	<0.001
**Human Opioid Death Rate**	-0.0266	-0.0416; -0.0115	0.001
**Human Opioid Death Rate**^**2**^	0.00114	-.000388; 0.00267	0.143
**Human Opioid Prescription Rate**	0.00548	0.00433; 0.00663	<0.001
**Human Opioid Prescription Rate**^**2**^	-0.0000344	-0.0000532; -0.0000156	<0.001
**Year**	-0.0491	-0.0600; -0.0382	<0.001
**Year**^**2**^	-0.00982	-0.0147; -0.00495	<0.001
**Weight**	-0.0285	-0.0311; -0.0259	<0.001
**Weight**^**2**^	0.000787	0.000669; 0.000905	<0.001
**Sex**			
Female	Referent		
Male	-0.0415	-0.0970; .0140	0.143
**Reproductive Status**			
Intact	Referent		
Neutered	-0.626	-0.686; -0.567	<0.001
Unknown	-0.323	-0.482; -0.164	<0.001
**Breed Class**			
Herding	Referent		
Hound	0.226	0.0893; 0.362	0.001
Non-Sporting	-0.0171	-0.164; 0.130	0.819
Sporting	-0.326	-0.452; -0.201	<0.001
Terrier	0.320	0.190; 0.449	<0.001
Toy	0.575	0.462; 0.688	<0.001
Working	-0.0662	-0.212; 0.0797	0.374
Foundation Stock Service	-0.423	-1.130; 0.284	0.241
Other	0.00830	-0.149; 0.165	0.917
**Source of Call**			
Public	Referent		
Veterinarian	0.389	0.330; 0.447	<0.001

^ǁ^Animal Poison Control Center

^†^Mixed logistic regression

### Mixed logistic regression

In the final mixed logistic regression model, there was a statistically significant association between the following independent variables and the odds of a call being related to an opioid poisoning: county-level human prescription rate, county-level human prescription rate^2^, year, year^2^, source of call (i.e., public or veterinarian), dog weight, dog weight^2^, dog age, dog age^2^, dog reproductive status, and breed class (Table 5). In addition, we also identified the following significant interaction effects: age with weight, age with weight^2^, and weight with breed class.

**Table 5:** Results of mixed multivariable multi-level logistic regression model examining the associations between each dog-level and community-level variable on the odds of a poisoning call to the APCC^ǁ^ being related to an opioid (2006–2014).

Parameter	Coefficient	95% Confidence Intervals	P-Value
**Dog Age**	-0.230	-0.261; -0.199	<0.001
**Dog Age**^**2**^	0.0156	0.0135; 0.0176	<0.001
**Human Opioid Prescription Rate**	0.0120	0.00788; 0.0161	<0.001
**Human Opioid Prescription Rate**^**2**^	-0.0000357	-0.0000553; -0.0000160	<0.001
**Year**	0.0273	-0.0135; 0.0682	0.190
**Year**^**2**^	-0.00759	-0.0125; -0.00266	0.003
**Dog Weight**	-0.000400	-0.0167; 0.0160	0.962
**Dog Weight**^**2**^	-0.0000733	-0.000371; 0.000225	0.630
**Sex**			
Female	Referent		
Male	-0.0349	-0.0912; 0.0215	0.225
**Reproductive Status**			
Intact	Referent		
Neutered	-0.182	-0.251; -0.112	<0.001
Unknown	-0.0779	-0.239; 0.0834	0.344
**Breed Class**			
Herding	Referent		
Hound	0.402	0.129; 0.675	0.004
Non-Sporting	0.158	-0.138; 0.453	0.296
Sporting	-0.167	-0.449; 0.115	0.246
Terrier	0.180	-0.0868; 0.447	0.186
Toy	0.655	0.396; 0.914	<0.001
Working	-0.0551	-0.392; 0.283	0.283
Foundation Stock Service	-0.462	-1.889; 0.964	0.526
Other	0.270	-0.0426; 0.582	0.091
**Source of Call**			
Public	Referent		
Veterinarian	0.332	0.273; 0.391	<0.001
**Interactions**			
**Age x Weight**	-0.00240	-0.00439; -0.000409	0.018
**Age x Weight**^**2**^	0.0000756	0.0000319; 0.000119	0.001
**Breed Class x Weight**			
Herding x Weight	Referent		
Hound x Weight	-0.0112	-0.0254; 0.00290	0.119
Non-Sporting x Weight	-0.0197	-0.0389; -0.000429	0.045
Sporting x Weight	-0.00578	-0.0173; 0.00575	0.326
Terrier x Weight	0.00851	-0.00439; 0.0214	0.196
Toy x Weight	-0.0425	-0.0650; -0.0200	<0.001
Working x Weight	-0.00281	-0.0157; 0.0100	0.668
Foundation Stock Service x Weight	0.00208	-0.0580; 0.0621	0.946
Other x Weight	-0.0194	-0.0350; -0.00371	0.015
**Variance Component**			
State	0.00460	0.00113; 0.0188	
County	0.0232	0.0109; 0.0493	

^ǁ^Animal Poison Control Center

The odds of a call being related to intoxication from an opioid were significantly greater for intact dogs than neutered dogs (OR = 1.22; 95% CI = 1.14–1.31; p-value<0.001) (Table 5). Additionally, the odds of a call being related to a poisoning with an opioid were significantly greater if the call came from a veterinarian rather than a member of the public (OR = 1.20; 95% CI = 1.12–1.29; p-value<0.001) (Table 5).

**Fig 2:**
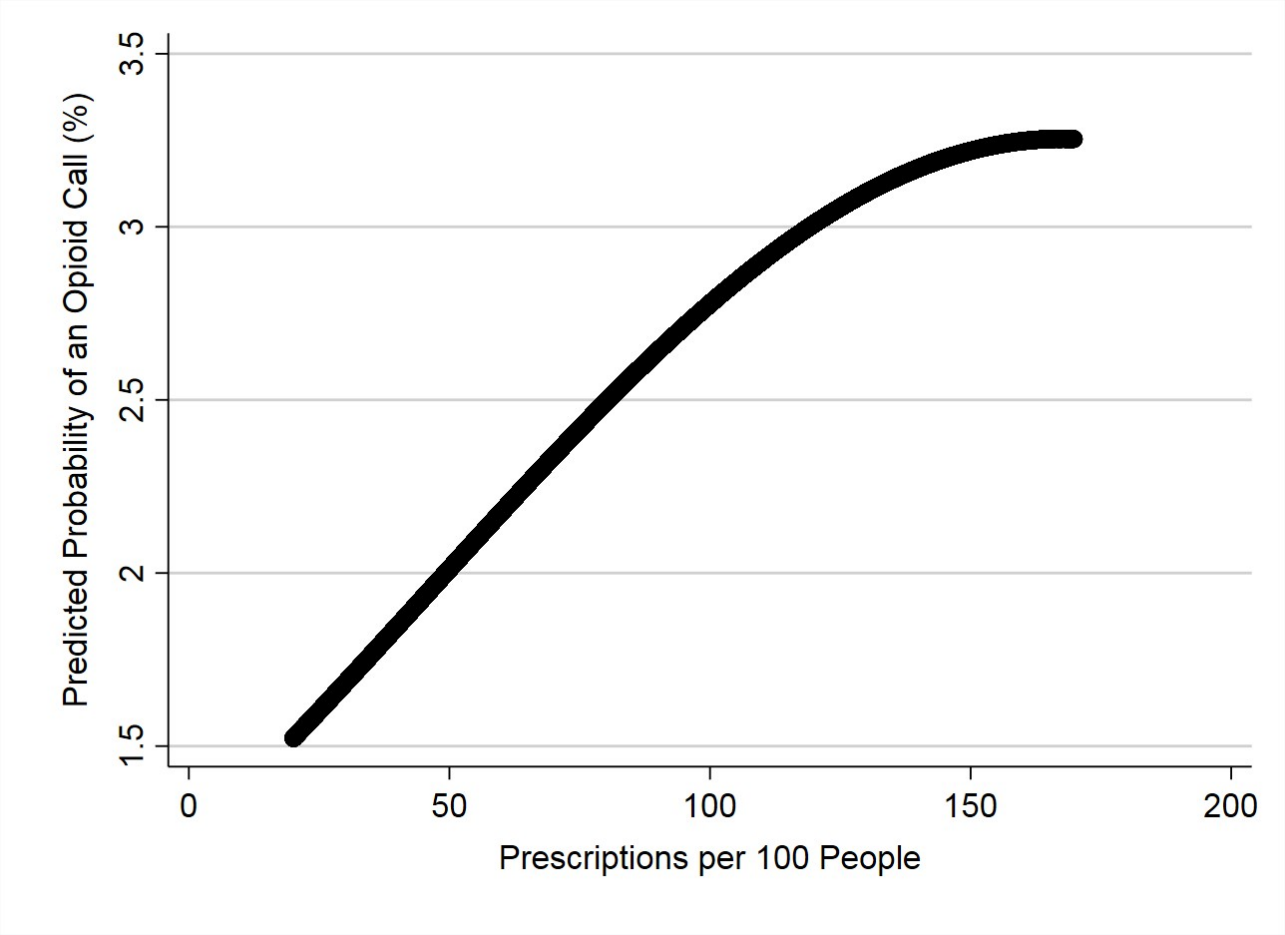
Predicted probability of opioid poisoning calls to the APCC* plotted against county-level prescription rate (2006–2014).

There was a significant quadratic relationship between the odds of a call being related to an opioid poisoning and the county-level human opioid prescription rate (Table 5). Initially, the odds increased with prescription rate and leveled off at approximately 150 prescriptions/100 people (Fig 2).

**Fig 3:**
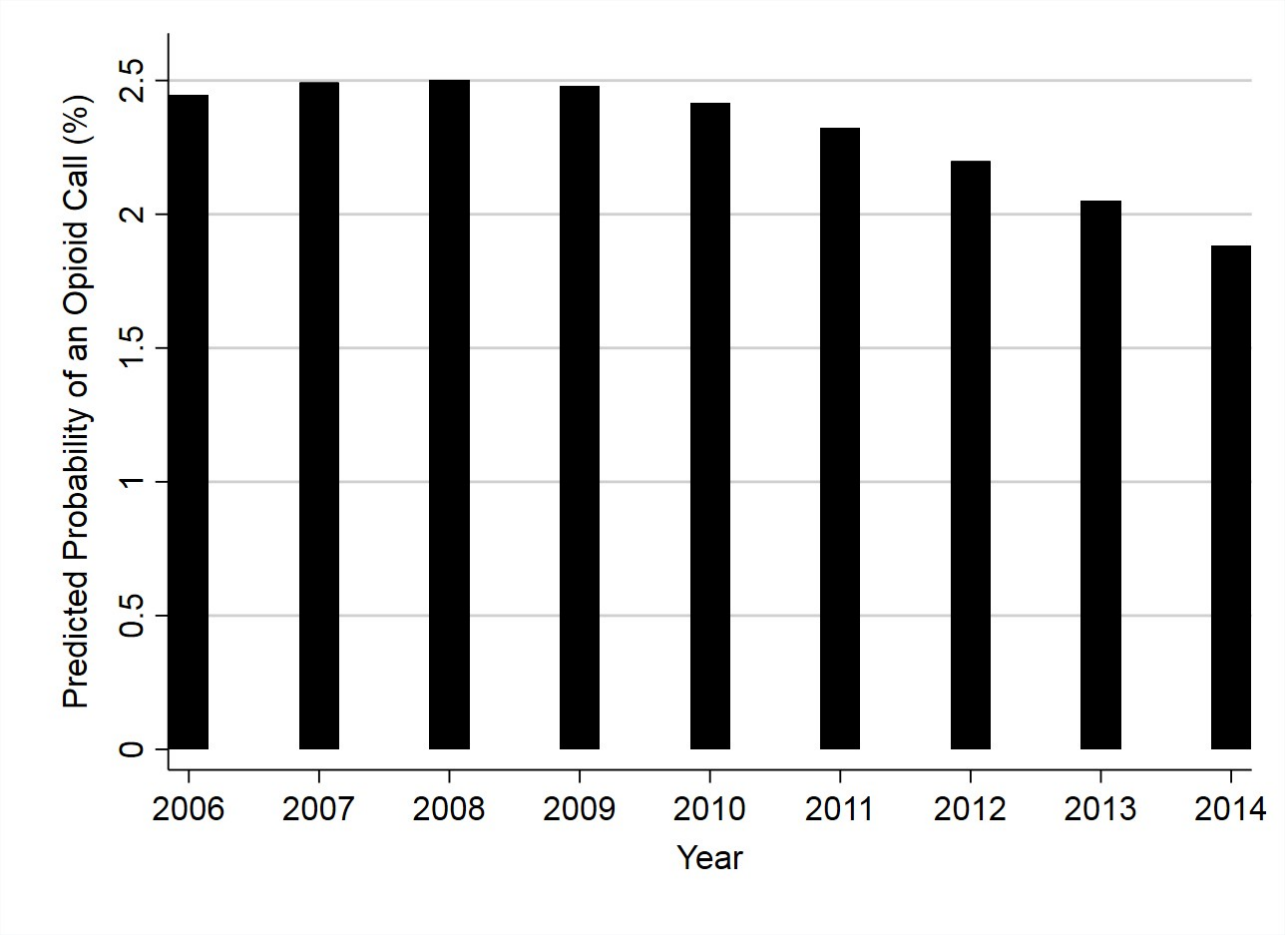
Predicted probability of opioid poisoning calls to the APCC* plotted against the year they were made (2006–2014).

There was also a significant relationship between year and the odds of a call being related to an opioid intoxication (Table 5). By year, the predicted probability of an opioid poisoning call initially increased from 2006–2008, but then decreased over the rest of the study period (Fig 3).

**Fig 4:**
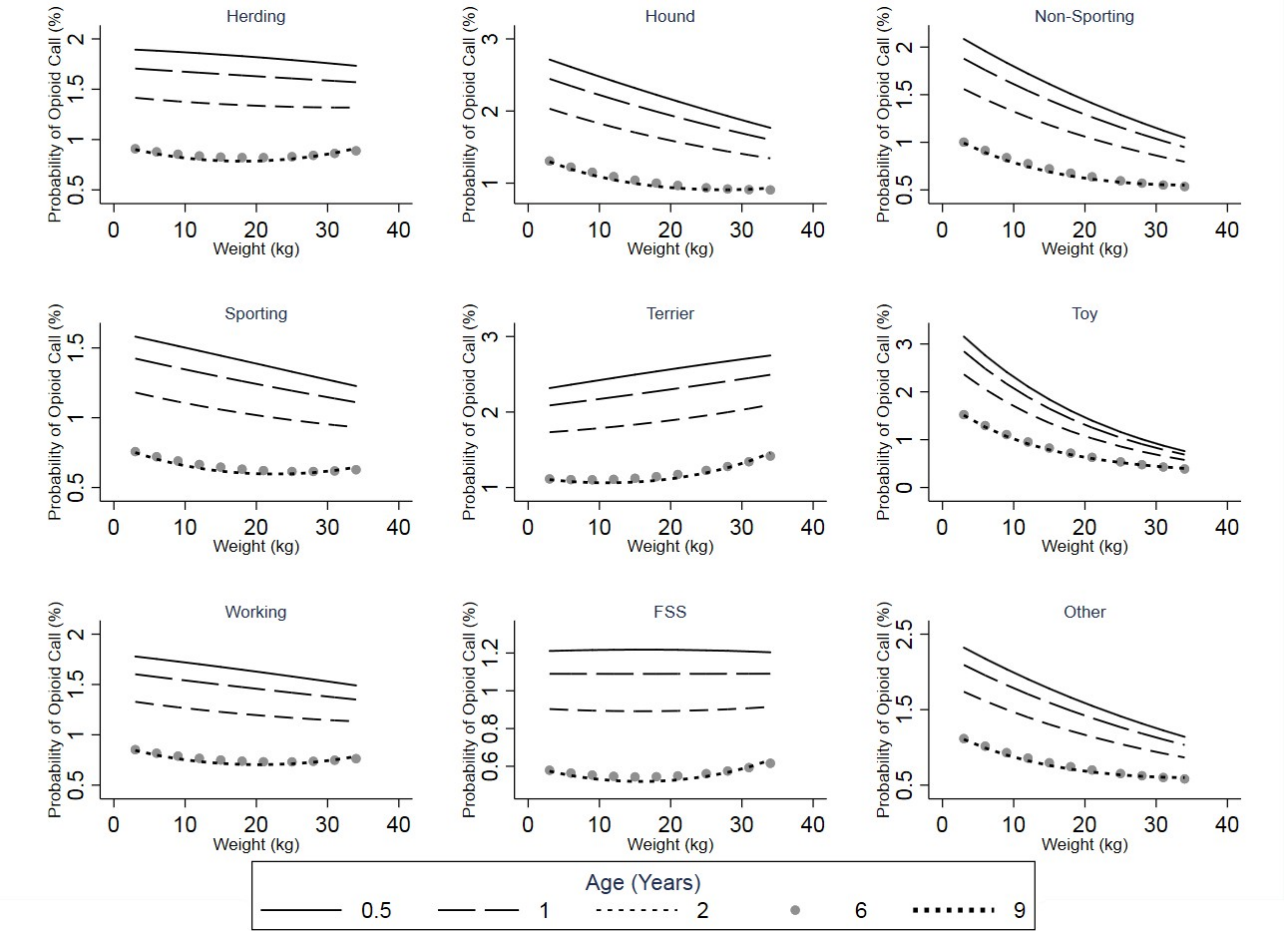
Predicted probabilities of opioid poisoning calls to the APCC* for each breed class plotted against weight for different age groups (2006–2014).

Statistically significant interactions were included in the final model; dog age interacted significantly with dog weight and weight^2^, while dog weight also interacted with breed class (Table 5). In all breed classes, as dog age increases, the predicted probability of an opioid poisoning call decreases, but this effect levels off for older animals (Fig 4). With all but two breed classes, dog weight has a non-linear negative relationship with the probability of an opioid poisoning call. Among the dogs in the terrier breed class, there is a non-linear positive association between dog weight and the probability of an opioid poisoning call (Fig 4), and for dogs in the FSS breed class, there appears to be no relationship between dog weight and this outcome (Fig 4). The effects of both dog age and weight varied by breed class.

Based on variance partition coefficients, 99.13%, 0.74%, and 0.13% of the variance was explained at the dog, county, and state-levels, respectively (Table 5). The best linear unbiased predictions (BLUPs) met the assumptions of both homoscedasticity and normality. One potential outlying observation was noted, but its removal did not impact the final model.

## Discussion

This study provides the first population-based national (U.S.) analysis to identify and quantify community and dog-level risk factors associated with opioid poisonings in dogs. Using call data provided by the ASPCA regarding dog poisonings reported to the APCC, we fit a multi-level logistic regression with random intercepts for county and state; and identified several dog and community-level factors that were associated with the odds of a call being related to canine exposure to an opioid.

### Dog-level variables

Intact dogs appear to be at greater odds than neutered dogs of having an opioid poisoning call to the APCC made on their behalf. This could suggest that changes in behaviour associated with neutering reduced the odds of opioid intoxication. However, it could also reflect differences in drug use between owners who elect to neuter their dogs and those who leave them intact.

Generally, there was a negative association between age/weight and the probability of an opioid poisoning call (Fig 4). Therefore, in most cases, the odds of a call being related to opioid poisoning were higher for smaller and younger dogs. This finding may reflect the way owners handle smaller/younger dogs; these younger and smaller dogs may live more closely and intimately with their owners leading to more exposure to drugs in the environment of these dogs. Younger dogs may also be more likely to ingest “novel” items. In addition, smaller/younger dogs would need a lower dose of an opioid to show clinical signs that might prompt a call to the APCC.

The probability of an opioid poisoning call initially increased from 2006 to 2008 where it peaked, and then subsequently fell until the end of the study in 2014. This temporal effect was noted even after controlling for county-level prescription rate. The reduction of dog opioid poisoning calls may be a hopeful metric, inferring opioid poisoning events in dogs are on a significant decline. However, this trend could be reflecting a growing awareness of the opioid epidemic [18]. With the appropriate interventions to avoid dog death more broadly known and opioid overdoses more recognizable, the resources required by the APCC to aid the public and veterinarians in these situations would be lowered. Similarly, the relative increase in non-opioid calls could account for this decline in opioid calls during the 2009–2014 period.

As a result of interventions aimed at reducing opioid prescription rates in the U.S.A., prescription rates as of 2012 have substantially decreased [14]. Yet, due to the shift in use from prescription opioids to more dangerous illicit opioids, human opioid death rates have doubled over the same time period as this study [19]. Although we controlled for prescription rate in our analysis, the reduction in the probability of a dog opioid call over time more closely follows trends of prescription rates than death rates. This phenomenon could be due to the increasing use of illicit opioids that cause more human deaths than prescription opioids [14]. Illicit opioids are likely better safeguarded and used immediately after obtained, making them likely less accessible to dogs than prescription opioids. With this reduced availability of prescription opioids to dogs, the shift from prescription opioids to illicit opioids may be more dangerous to humans with fewer accidental intoxications involving dogs.

The odds of an opioid call coming from a veterinarian compared to the public is significantly greater. This finding may reflect a lack of willingness or fear of owners to report poisonings that may have legal implications associated with illegal drug possession.

Based on our analysis, there was no evidence to suggest the sex of the dog had a significant impact on the odds of an opioid poisoning call being made to the APCC or interacted with other animal-level characteristics (e.g., reproductive status).

### Human use variables

By merging state-level opioid-related death rates and county-level human prescription rates with the AnTox database, we were able to study the effects of these community-level predictor variables on the odds of an opioid poisoning call to the APCC. The results of our analysis showed a strong positive association between county-level human opioid prescription rate and the probability of an opioid poisoning call. This association could infer the following: dogs in counties with higher prescription rates are at a higher risk of being poisoned by an opioid, or owners of dogs in these communities are more willing to report dog opioid poisonings to the APCC.

There was insufficient evidence to suggest that human opioid death rate at the state-level had any bearing on the probability of an opioid poisoning call coming to the APCC. State-level data may not be granular enough to sufficiently reflect the variation among regions in the rate of opioid-related deaths.

## Conclusion

Although we identified community and dog-level characteristics that impacted opioid calls to the APCC, studies of this nature in various jurisdictions and scales are essential to strengthen our understanding of these poisoning events. As the human opioid-related death rate caused by illegally obtained opioids continues to increase and human opioid prescription rates continue to decrease, it would be valuable to determine the proportion of dogs being poisoned by legally or illegally obtained opioids. Additionally, geospatial studies are needed to identify particular areas of higher opioid poisoning risk, so those regions can be targeted for interventions including increased public and veterinary education.

A variety of potential systematic biases should be recognized when considering our results. First, all variables are self-reported by veterinarians and the public, so information bias needs to be considered. In addition, there is the potential for selection bias since reporting is voluntary. For instance, it is possible that dogs exposed to opioids die before a call is made to the APCC. Selection bias would occur if the occurrence of dog death before a call is made differs by exposure status. Similarly, services from the APCC costs 65 USD per case, which could inherently result in the associations measured among people using the service being systematically different from those who do not or cannot afford to use the service (i.e. non-response bias).

In theory, the most valuable information in predicting the likelihood of opioid poisoning in dogs would concern the use and/or presence of legal/illicit opioids in the same household as the dogs. However, these data are not available, and sampling this information directly from dog owners without introducing severe bias would be extremely difficult. Consequently, the use of county vs. household data concerning opioid prescription is an imperfect ecological variable but is an obtainable source of information concerning potential animal exposures.

This study suggests that generally a dog being smaller, younger, intact, or residing in counties with high prescription rates increases their odds of having an opioid call made on their behalf, and that those calls are more likely to be made by a veterinarian than the public. An awareness of dog characteristics that place members of this population at higher risk for opioid poisonings may help mitigate further harm to pet dogs where legal and illegal opioids are being consumed for legitimate and illicit purposes. Veterinarians and poison control centers responding to animal poisonings may benefit from knowledge of the opioid use patterns in human populations and their impact on dogs. This study also infers that opioid calls are decreasing in total and proportionally to all other toxicant calls, suggesting that even though the opioid epidemic is escalating for humans [19], the situation may be improving for canines. We hope that this study helps to further characterize the opioid epidemic [18] as a whole and brings awareness of the spill-over effect of human opioid use on pet dogs to the public and veterinary community.

## References

[1] Wang H, Naghavi M, Allen C, Barber RM, Bhutta ZA, Carter A, et al Global, regional, and national life expectancy, all-cause mortality, and cause- specific mortality for 249 causes of death, 1980–2015: a systematic analysis for the Global Burden of Disease Study 2015. The Lancet. 2016; 388: 1459–1544. 10.1016/S0140-6736(16)31012-1 PMC5388903 27733281

[2] Rudd RA, Seth P, David F, Scholl L. Increases in Drug and Opioid-Involved Overdoses in the United States, 2010–2015. MMWR Morbidity and Mortality Weekly Report. 2016;65: 1445–1452. 10.15585/mmwr.mm655051e1 28033313

[3] Centers for Disease Control and Prevention. Vital signs: overdoses of prescription opioid pain relievers–United States, 1999–2008. MMWR Morbidity and Mortality Weekly Report. 2011;60: 1487 22048730

[4] Martin T, Rocque M. Accidental and non-accidental ingestion of methadone and buprenorphine in childhood: a single center experience, 1999–2009. Current Drug Safety. 2011;6: 12–16. 10.2174/157488611794480034 21047302

[5] Center for Behavioral Health Statistics and Quality. Key substance use and mental health indicators in the United States: Results from the 2015 National Survey on Drug Use and Health. 2017. E-print. Available from: http://www.samhsa.gov/data/. (HHS Publication No. SMA 16–4984, NSDUH Series H-51). Cited 25 June 2019.

[6] Burghardt LC, Ayers JW, Brownstein JS, Bronstein AC, Ewald MB, Bourgeois FT. Adult Prescription Drug Use and Pediatric Medication Exposures and Poisonings. Pediatrics. 2013;132: 18–27. 10.1542/peds.2012-2978 23733792 PMC4074615

[7] Bailey JE, Campagna E, Dart RC. The Underrecognized Toll of Prescription Opioid Abuse on Young Children. Annals of Emergency Medicine. 2009;53: 419–424. 10.1016/j.annemergmed.2008.07.015 18774623

[8] Lovegrove MC, Mathew J, Hampp C, Governale L, Wysowski DK, Budnitz DS. Emergency Hospitalizations for Unsupervised Prescription Medication Ingestions by Young Children. Pediatrics. 2014;134: 1009–1016. 10.1542/peds.2014-0840 PMC4651431 25225137

[9] Gaither JR, Leventhal JM, Ryan SA, Camenga DR. (2016) National trends in hospitalizations for opioid poisonings among children and adolescents, 1997 to 2012. JAMA Pediatrics. 2016;170: 1195–1201. 10.1001/jamapediatrics.2016.2154 27802492 PMC7245935

[10] Gfeller RW, Messonnier SW. Handbook of Small Animal Toxicology and Poisonings. 2^nd^ ed. St. Louis: Mosby; 2004.

[11] Meola SD, Tearney CC, Haas SA, Hackett TB, Mazzaferro EM. (2012). Evaluation of trends in marijuana toxicosis in dogs living in a state with legalized medical marijuana: 125 dogs (2005–2010). Journal of Veterinary Emergency and Critical Care. 2012;22: 690–696. 10.1111/j.1476-4431.2012.00818.x 23216842

[12] Wang GS, Roosevelt G, Heard K. Pediatric marijuana exposures in a medical marijuana state. JAMA Pediatrics. 2013;167: 630–633. 10.1001/jamapediatrics.2013.140 23712626

[13] Berny P, Caloni F, Croubels S, Sachana M, Vandenbroucke V, Davanzo F, Guitart R. Animal poisoning in Europe. Part 2: Companion animals. Veterinary Journal. 2010;183: 255–259. 10.1016/j.tvjl.2009.03.034 19553146

[14] Centers of Disease Control and Prevention. 2018 Oct 3 [cited 25 June 2019]. In: US opioid prescribing rate maps [Internet]. Atlanta: Available from: https://www.cdc.gov/drugoverdose/maps/rxrate-maps.html.

[15] Centers of Disease Control and Prevention. 2019 Jun 25 [cited 25 June 2019]. In: Wide-ranging online data for epidemiologic research (WONDER) [Internet]. Atlanta: Available from: https://wonder.cdc.gov/.

[16] Dohoo IR, Martin SW, Stryhn H. Veterinary epidemiologic research. 2nd ed Charlottetown: AVC Inc; 2009.

[17] Wasserstein RL, Schirm AL, Lazar NA. Moving to a World Beyond “p< 0.05.”. The American Statistician. 2019;73: 1–19.

[18] Manchikanti L, Fellows B, Janata JW, Pampati V, Grider JS, Boswell MV. Opioid epidemic in the United States. Pain physician. 2012;15: 9–38 22786464

[19] National Institute of Health. 2019 Jan [cited 25 June 2019]. In: Overdose Death Rates [Internet]. Bethesda: Available from: https://www.drugabuse.gov/related-topics/trends-statistics/overdose-death-rates.

